# Integrated analysis of long non-coding RNA and mRNA expression in different colored skin of koi carp

**DOI:** 10.1186/s12864-019-5894-8

**Published:** 2019-06-21

**Authors:** Mingkun Luo, Lanmei Wang, Haoran Yin, Wenbin Zhu, Jianjun Fu, Zaijie Dong

**Affiliations:** 1 Wuxi Fisheries College, Nanjing Agricultural University, Wuxi, 214081 Jiangsu China; 2 Freshwater Fisheries Research Center of Chinese Academy of Fishery Sciences, Key Laboratory of Freshwater Fisheries and Germplasm Resources Utilization, Ministry of Agriculture and Rural Affairs, Wuxi, 214081 Jiangsu China

**Keywords:** LncRNA, mRNA, Skin color, Function annotation, Koi carp, Illumina sequencing

## Abstract

**Background:**

Long non-coding RNAs (lncRNAs) perform crucial roles in biological process involving complex mechanisms. However, information regarding their abundance, characteristics and potential functions linked to fish skin color is limited. Herein, Illumina sequencing and bioinformatics were conducted on black, white, and red skin of Koi carp (*Cyprinus carpio* L.).

**Results:**

A total of 590,415,050 clean reads, 446,614 putative transcripts, 4252 known and 72,907 novel lncRNAs were simultaneously obtained, including 92 significant differentially expressed lncRNAs and 722 mRNAs. *Ccr_lnc5622441* and *Ccr_lnc765201* were up-regulated in black and red skin, *Ccr_lnc14074601* and *Ccr_lnc2382951* were up-regulated in white skin, and premelanosome protein a (*Pmela*), *Pmelb* and tyrosinase (*Tyr*) were up-regulated in black skin. The expression patterns of 18 randomly selected differentially expressed genes were validated using the quantitative real-time PCR method. Moreover, 70 lncRNAs acting on 107 target mRNAs in *cis* and 79 lncRNAs acting on 41,625 target mRNAs in *trans* were investigated. The resulting co-expression networks revealed that a single lncRNA can connect with numerous mRNAs, and vice versa. To further reveal their potential functions, Gene Ontology (GO) terms and Kyoto Encyclopedia of Genes and Genomes (KEGG) pathways were analyzed, and membrane, pigment cell development, cAMP signaling, melanogenesis and tyrosine metabolism appear to affect skin pigmentation. Additionally, three lncRNAs (*Ccr_lnc142711*, *Ccr_lnc17214525* and *Ccr_lnc14830101*) and three mRNAs (*Asip*, *Mitf* and *Tyr*) involved in the melanogenesis pathway were investigated in terms of potential functions in embryogenesis and different tissues.

**Conclusions:**

The findings broaden our understanding of lncRNAs and skin color genetics, and provide new insight into the mechanisms underlying lncRNA-mediated pigmentation and differentiation in Koi carp.

**Electronic supplementary material:**

The online version of this article (10.1186/s12864-019-5894-8) contains supplementary material, which is available to authorized users.

## Background

Non-coding RNAs (ncRNAs), which are not translated into proteins but play vital roles in various biological processes, are receiving increasing attention [1]. Among them, long non-coding RNAs (lncRNAs), defined as transcripts longer than 200 nucleotides and lacking protein-coding capacity, have emerged as essential mediators in numerous biological functions [2]. LncRNAs positively regulate gene expression at different levels, including transcriptional and posttranscriptional regulation, and chromosome remodeling [3]. Through these actions, lncRNAs typically function in protein localization, telomere replication and RNA interference [4]. With next-generation high-throughput RNA sequencing techniques and computational analysis, thousands of lncRNA transcripts have been identified through systematic sequencing of full-length cDNA libraries in many species, such as *Homo sapiens* [5], *Rattus norvegicus* [6], *Salmo salar* [7] and *Danio rerio* [8].

Recently, lncRNAs have also been suggested to perform a crucial role in regulating skin. Researchers have discovered several lncRNAs associated with skin biology such as *BANCR*, *ANCR*, *U1 RNA* and *PRINS* [9]. A series of well-known oncogenes including *H19*, *H19-as*, *Foxn2-as* and *Hottip* function in vitamin D receptor protection against skin cancer formation by helping to maintain the balance between oncogenic and tumor-suppressing lncRNAs [10]. Ren et al. identified 1336 lncRNAs in goat fetal skin and investigated significant differences in gene architecture, expression levels, and impact on target genes in *cis* and *trans* [11]. Weikard et al. demonstrated a complex transcript pattern for bovine skin and identified 4365 potential intergenic lncRNAs in bulls with a piebald phenotype [12]. To our knowledge, there have been no reports describing the involvement of lncRNAs in skin color pigmentation and differentiation in fish.

Koi carp, a colorful variant of common carp (*Cyprinus carpio* L.), is one of the most important ornamental fish worldwide with great economic value [13]. Some individuals display fascinating skin color patterns that play important roles in numerous biological processes including mate-choice, camouflage, and perception of threatening behavior [14, 15]. Skin color regulation in fish is a complicated process, linked to various cellular, genetic, nutritional, and environmental factors [16]. In Koi carp, much attention has been paid to skin color regulation, including the genetics of pigment patterning [17], cloning of color-related genes, functional analysis [18, 19], environmental factors [20], nutrition [21] and transcriptome analysis [22]. However, lncRNA-mediated regulatory mechanisms related to skin color have not been reported.

In our previous study, we performed small RNA sequencing (sRNA-Seq) on three skin colors (black, white and red) in Koi carp using Illumina sequencing. We screened 164 differentially expressed miRNAs and identified several key miRNAs related to pigment regulation including *miR-196a*, *miR-200b*, *miR-125b* and *miR-202* [23]. In the present study, we conducted a high-throughput sequencing strategy to screen expression of lncRNAs and mRNAs in these three skin colors. Differentially expression patterns were validated using qRT-PCR to confirm the results of RNA-seq. Putative target mRNAs of lncRNAs in *cis* and *trans* were predicted, and we selected candidate lncRNAs and mRNAs that are highly likely affect skin pigmentation to construct coding/non-coding gene co-expression diagrams. Moreover, we also analyzed the Gene Ontology (GO) terms and Kyoto Encyclopedia of Genes and Genomes (KEGG) pathways to excavate the function roles of genes in the determination of skin color. Spatial and temporal expression patterns of some lncRNAs and mRNAs involved in the melanogenesis pathway were further analyzed, indicating that these genes have strict tissue specificity during skin development and pigmentation. Our findings provide an invaluable resource for understanding the genetics of skin color in fish, and expand our knowledge on lncRNA biology in general in Koi carp.

## Results

### Identification of lncRNAs in koi carp skin

**Fig. 1:**
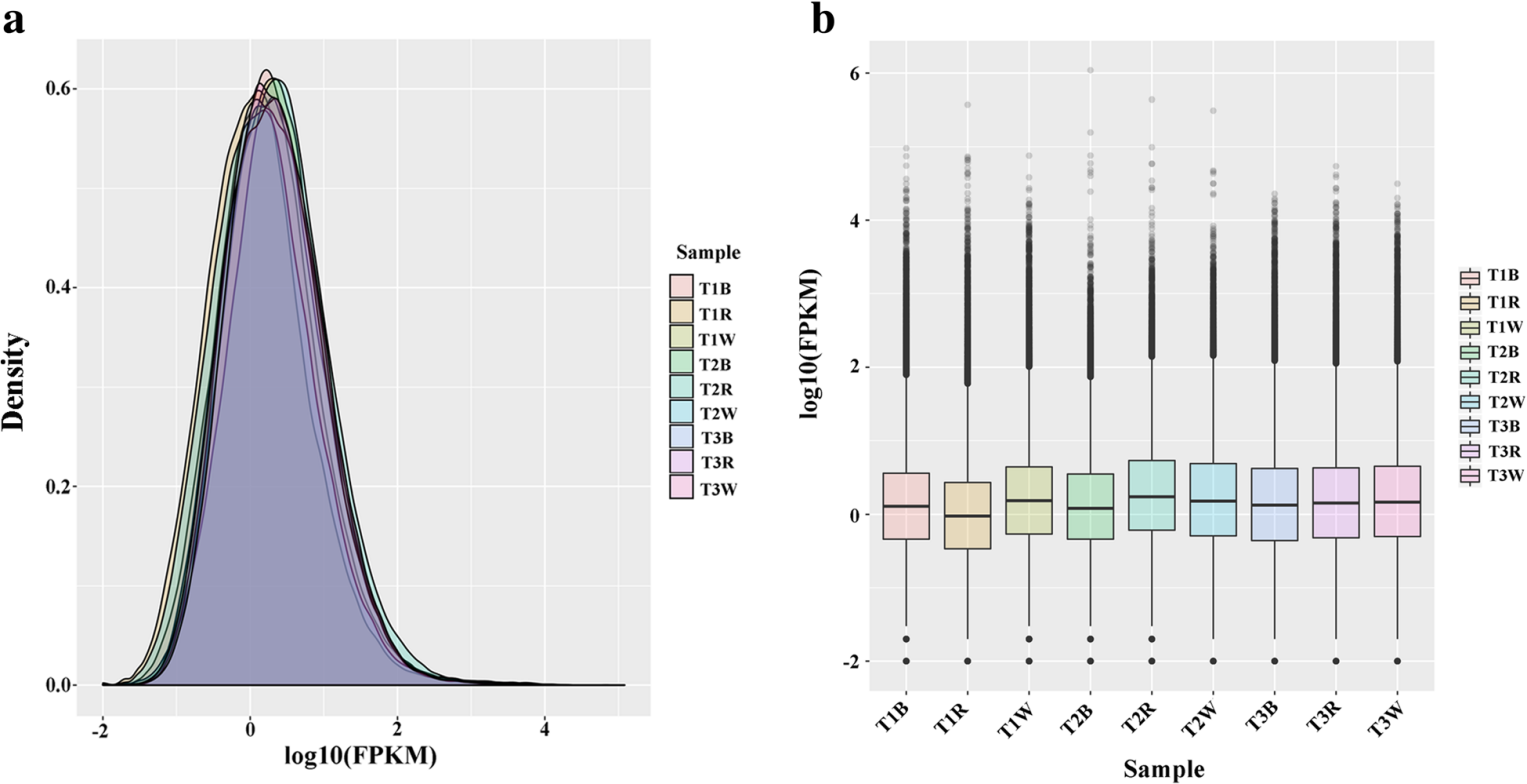
**a** Density plot showing the distribution of each sample. **b** Box plot showing the distribution of maximum, minimum and percentile values for normalized signals of each sample

A total of 612,253,232 raw reads were excavated from the nine sequencing libraries. After discarding adaptor and low-quality sequences, 590,415,050 clean reads remained, and the percentage of clean reads among raw tags in each library ranged from 95.83–96.95% (Additional file 2). The quality of transcript expression level measurements was checked by density plots for each sample (Fig. 1a) and the distribution of maximum, minimum, and percentile values for normalized signals for each sample (Fig. 1b). Subsequently, clean reads were mapped to the *C. carpio* v3.0 reference genome, resulting in 446,614 putative transcripts (Table 1). Several filtering steps were applied to screen lncRNAs in the transcript list by removing protein-coding transcripts, pseudogenes, and other classes of non-coding RNAs including ribosomal RNA (rRNA), microRNA (miRNA), transfer ribonucleic acid (tRNA). We identified 77,159 expressed lncRNAs, of which 4252 (5.5%) were previously annotated and 72,907 (94.5%) were novel lncRNAs (Additional file 3).

**Table 1:** Summary of Koi carp sequencing data

Item	Value
Total number of sequences	698,058
Total number of genes	446,614
Total base of sequences (Mb)	644
Maximum sequence length (bp)	17,984
Minimum sequence length (bp)	200
Average sequence length (bp)	922.82
Median contig length (bp)	616
N50 (bp)	1328
Percent GC (%)	43.55

### Expression profiling of lncRNAs and mRNAs

**Fig. 2:**
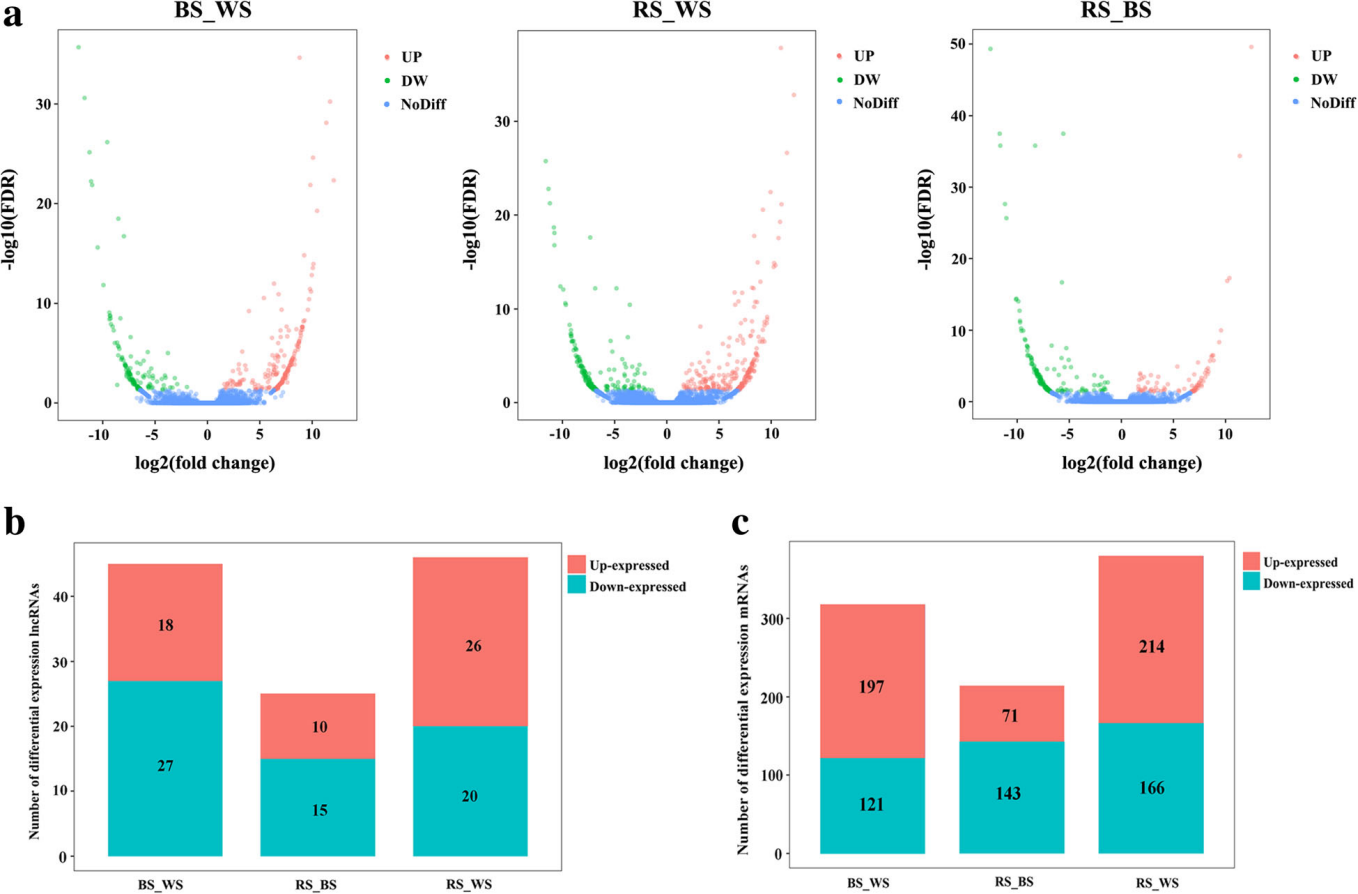
Differentially expressed genes in the three pairwise comparison groups. **a** Volcano plots of all genes in the three pairwise comparisons. Blue, red and green dots represent non-significant, up- and down-regulated genes, respectively; **b** Brown and cyan indicate the number of significant up- and down-regulated lncRNAs, respectively; **c** Brown and cyan indicate the number of significant up- and down-regulated mRNAs, respectively

A total of 722 mRNAs and 92 lncRNAs were identified as significant, and volcano plots of three pairwise comparisons were plotted to reveal expression trends (Fig. 2a). Among differentially expressed (DE) lncRNAs, 18 were up-regulated and 27 were down-regulated in BS compared with WS; 10 lncRNAs were up-regulated and 15 were down-regulated in RS compared with BS; and 26 lncRNAs were up-regulated and 20 were down-regulated in RS compared with WS (Fig. 2b). Furthermore, among DE mRNAs, 197 mRNAs were up-regulated and 121 were down-regulated between BS and WS, 71 mRNAs were up-regulated and 143 were down-regulated between RS and BS, and 214 mRNAs were up-regulated and 166 were down-regulated between RS and WS (Fig. 2c). The proportion of significantly DE lncRNAs and mRNAs reflects their specific functions, and detailed lists of all genes are provided in Additional file 4.

**Fig. 3:**
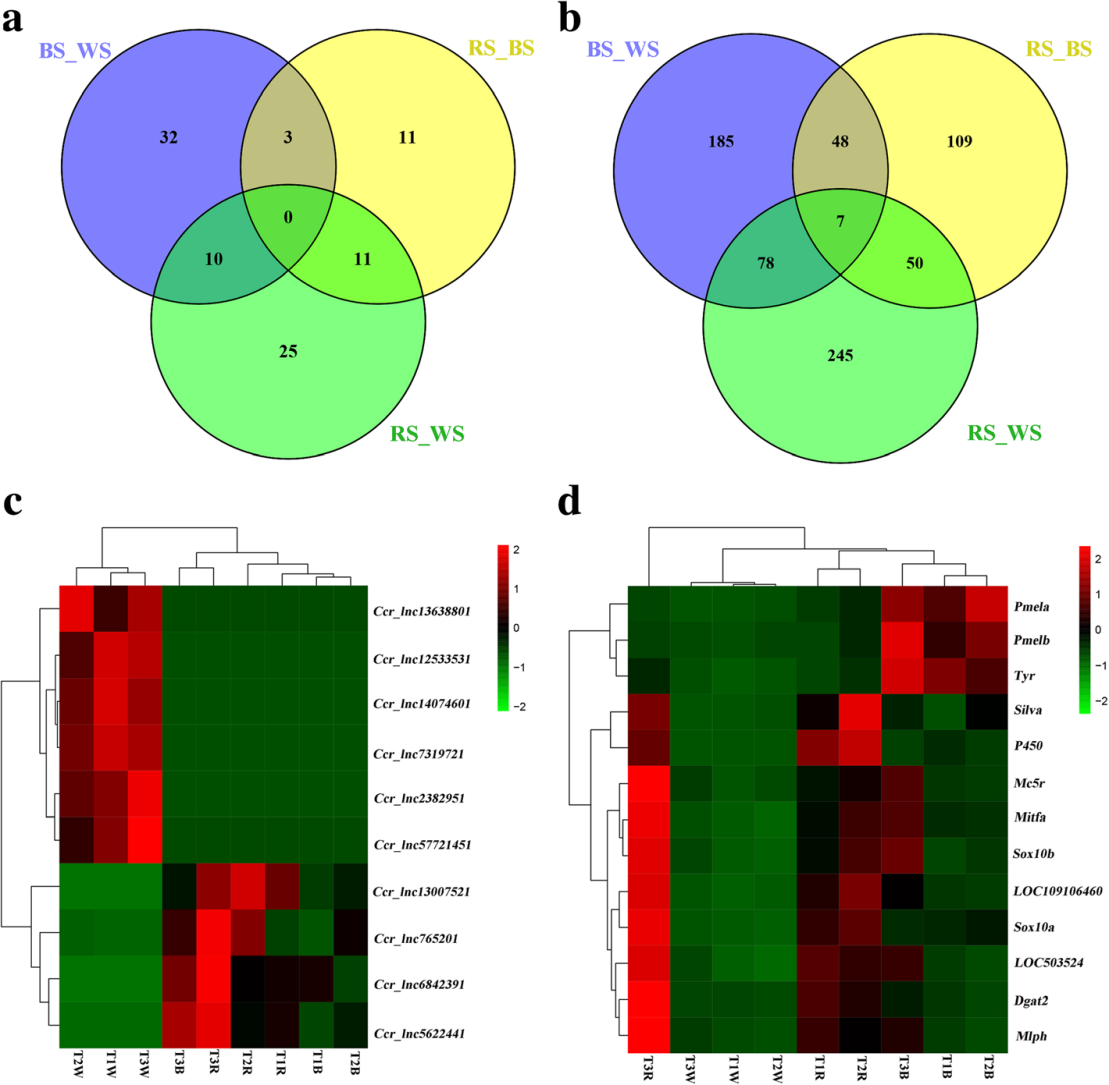
**a** Venn diagram of differentially expressed lncRNAs in the three pairwise comparison groups; **b** Venn diagram of differentially expressed mRNAs in the three pairwise comparison groups; **c** Heatmaps and hierarchical clustering of 10 selected lncRNAs. **d** Heatmaps and hierarchical clustering of 13 randomly selected mRNAs. High to low expression is colored green to red

Venn 2.1 (http://bioinfogp.cnb.csic.es/tools/venny/index.html) was employed to further visually compare gene expression. Among the lncRNAs, 11 known and 81 novel lncRNAs were identified, and 10 lncRNAs were DE in both RS and BS groups compared with the WS group (Fig. 3a). Meanwhile, 722 mRNAs including seven overlapping sequences were also identified, and 85 mRNAs were DE in both RS and BS groups compared with the WS group (Fig. 3b). Ten lncRNAs and 13 mRNAs (randomly selected from the 85 mRNAs) were chosen for plotting heatmaps. Hierarchical clustering allowed us to hypothesize relationships among these genes, and the 10 DE lncRNAs (Fig. 3c) and 13 mRNAs (Fig. 3d) presented different expression patterns. For example, *Ccr_lnc5622441*, *Ccr_lnc765201*, *Ccr_lnc6842391* and *Ccr_lnc13007521* were up-regulated in RS and BS groups compared with the WS group, and *Pmela*, *Pmelb* and *Tyr* were up-regulated in BS compared with RS and WS.

**Fig. 4:**
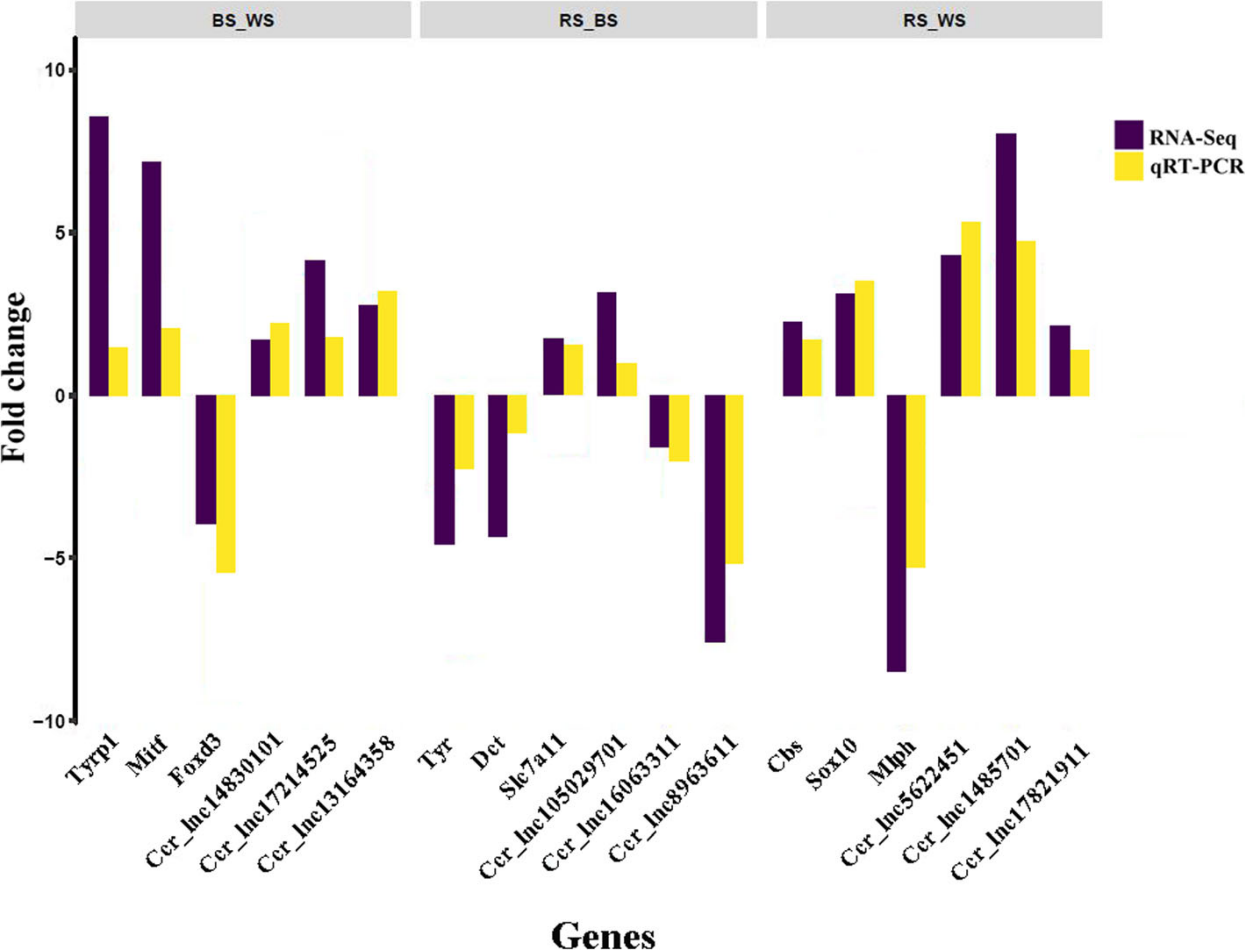
Relative expression levels of genes measured using quantitative real-time PCR (qRT-PCR) and Illumina sequencing (RNA-seq) in the three comparative groups

To further confirm the reliability and validity of the sequencing data, we randomly selected nine significant DE mRNAs and nine DE lncRNAs for analysis by qRT-PCR. The result showed that the qRT-PCR expression patterns were in complete agreement with the RNA-seq results, indicating that the RNA-seq data was reliable (Fig. 4). For example, based on the RNA-seq results, expression of *Sox10* in RS was almost 2.8 times higher than in WS, compared with a 3.2-fold difference according to qRT-PCR.

### GO and KEGG enrichment of differentially expressed mRNAs

**Fig. 5:**
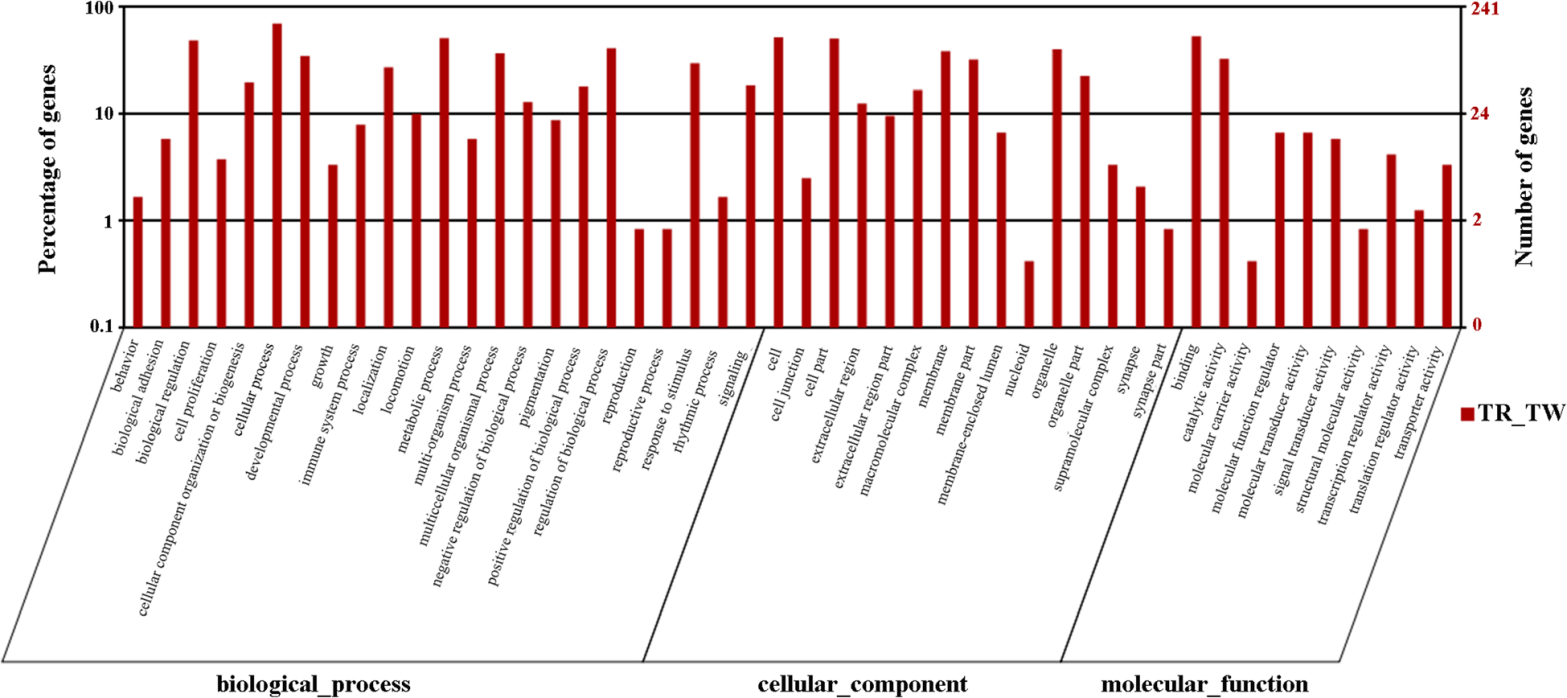
Gene Ontology (GO) enrichment analysis of significant differentially expressed mRNAs in RS and WS groups

To further explore the potential functions of 722 significant DE mRNAs, we analyzed the associated functions using the GO database. As shown in Figs. 5, 48 terms were assigned including 23 (47.92%) biological process, 15 (31.25%) cellular component and 10 (20.83%) molecular function categories in RS vs. WS. The results of all comparisons are presented in Additional file 5: Figure S3 and S4). The results showed that the most highly enriched terms were membrane (cellular component), binding (molecular function), and cellular process (biological process). Moreover, we identified some differently enriched terms in the three comparisons. For example, melanosome in cellular component and melanosome organization in biological process were more highly enriched in BS_WS and RS_BS groups than the RS_WS group. Detailed results are provided in Additional file 5.

In the KEGG pathway analysis of DE mRNAs, *p*-value denotes significance, with lower *p*-values indicating greater significance (the *p*-value cut-off determining significance was 0.05). The top 10 enriched pathways are presented in Table 2. Interestingly, some pathways, including melanogenesis and tyrosine metabolism, were more highly enriched in RS_BS and BS_WS groups than RS_WS, while tight junction was more highly enriched in the RS_WS comparisons. All 206 pathways identified in the three comparisons are presented in Additional file 5.

**Table 2:** The top ten KEGG pathways related to differentially expressed mRNAs in the three comparative groups

RS_WS group	RS_BS group	BS_WS group
Tight junction	Melanogenesis	Melanogenesis
Osteoclast differentiation	Tyrosine metabolism	Tyrosine metabolism
Pathways in cancer	Hypertrophic cardiomyopathy (HCM)	Neuroactive ligand-receptor interaction
Neuroactive ligand-receptor interaction	Dilated cardiomyopathy	Ether lipid metabolism
Fat digestion and absorption	Cardiac muscle contraction	Glycerophospholipid metabolism
Retinol metabolism	Circadian entrainment	Osteoclast differentiation
Viral myocarditis	Adrenergic signaling in cardiomyocytes	Linoleic acid metabolism
Cardiac muscle contraction	Fatty acid degradation	alpha-Linolenic acid metabolism
Cytokine-cytokine receptor interaction	Fatty acid metabolism	Retinol metabolism
Ras signaling pathway	Cell adhesion molecules (CAMs)	Arachidonic acid metabolism

### *Cis* and *trans* role of lncRNAs

**Fig. 6:**
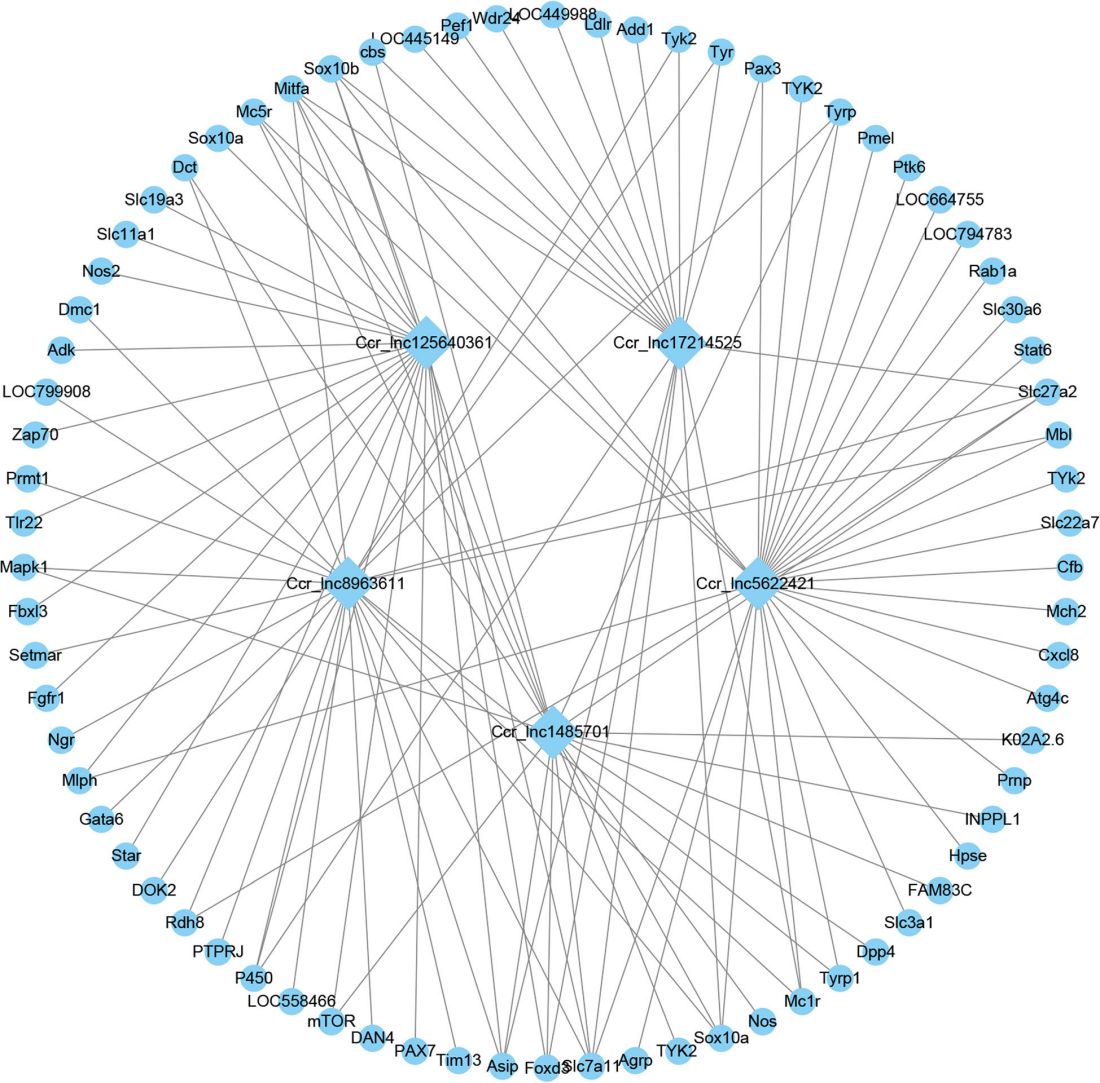
Networks analysis of putative interactions between five selected lncRNAs and *trans-*regulated mRNAs related to the pigmentation process. Square and circular nodes represent lncRNAs and protein-coding genes, respectively

The potential *cis* and *trans* targets of lncRNAs were predicted to investigate the functions of 92 DE lncRNAs. Regarding *cis* action, 70 DE lncRNAs corresponded to 107 protein-coding genes (Additional file 6). Among them, the skin color-related genes such as *Wnt3a*, *Slc45a2* and *Mitf* located near the *Ccr_lnc8247101*, *Ccr_lnc13638801* and *Ccr_lnc17821911* loci, respectively. GO term and KEGG pathway analysis were also conducted on 107 *cis* lncRNA targets to explore their functions (Additional file 6). A total of 41,625 interactions were identified for *trans* actions between 79 lncRNAs and protein-coding genes (Additional file 7). The interaction networks are quite complex, with some trans-regulation relationships evident (Fig. 6). For example, some mRNAs (*Mitfa*, *Slc7a11* and *Sox10a*) appear to be regulated by multiple lncRNAs (*Ccr_lnc8963611*, *Ccr_lnc1485701* and others), and one lncRNA (*Ccr_lnc5622421*) can target many mRNAs (*Tyk2*, *Mc1r*, *Tyrp1* and others).

**Fig. 7:**
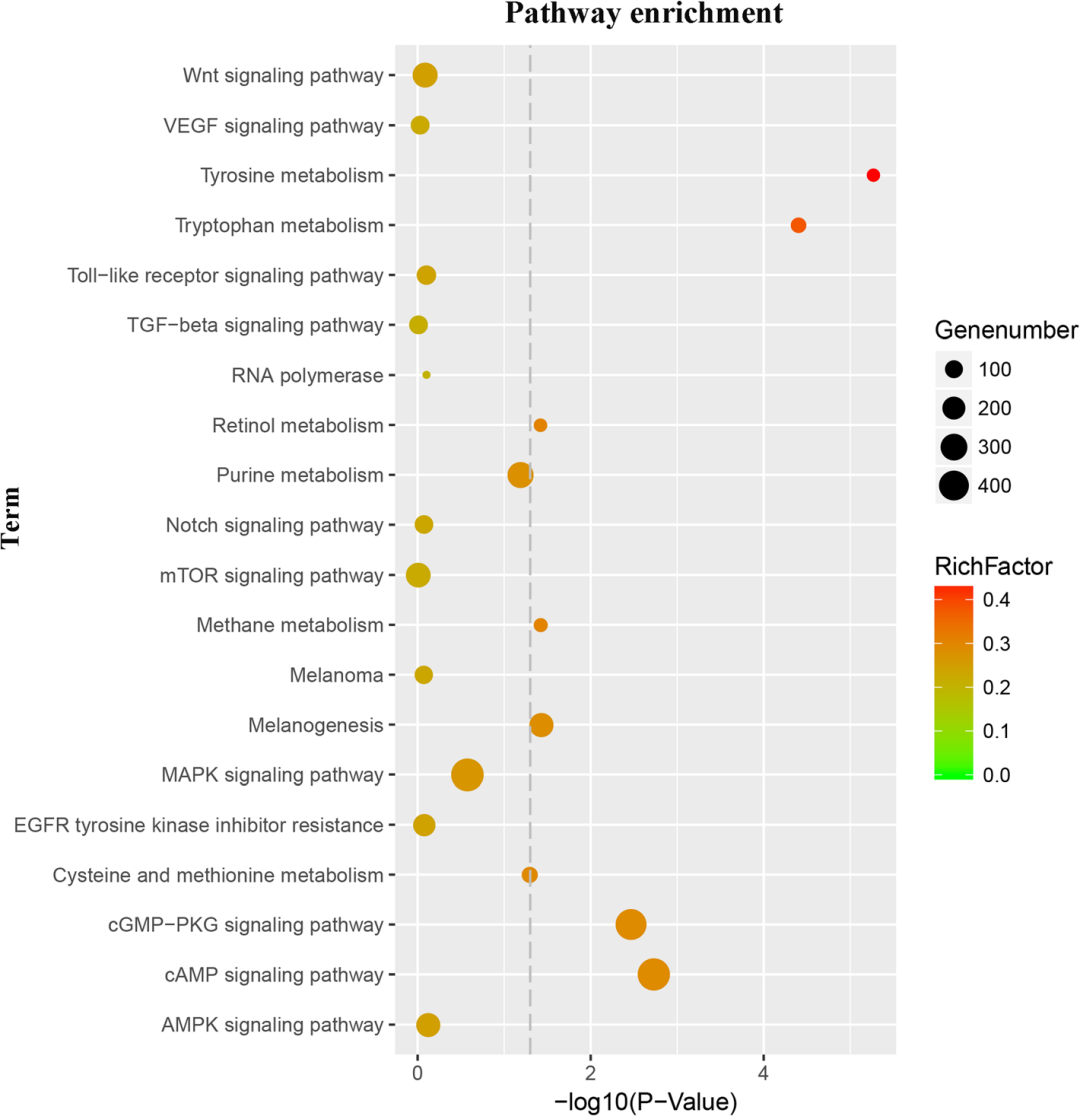
Predicted pigmentation-related pathways of *trans*-target mRNAs based on Kyoto Encyclopedia of Genes and Genomes (KEGG) pathway analysis. Gene number, number of target genes in each pathway; Rich factor, ratio of the number of target genes divided by the total number of genes in each pathway

We further analyzed these *trans*-targeted mRNA functions, and 6139 GO terms and 355 KEGG pathways were identified (Additional file 7). These include a number of terms related to pigment regulation, such as protein tyrosine kinase activity, retinal pigment epithelium development, pigment cell development, and melanin metabolic process. Importantly, we also identified some pathways related to pigmentation, including cAMP, Wnt, MAPK signaling pathways, melanogenesis, and tyrosine metabolism (Fig. 7).

**Fig. 8:**
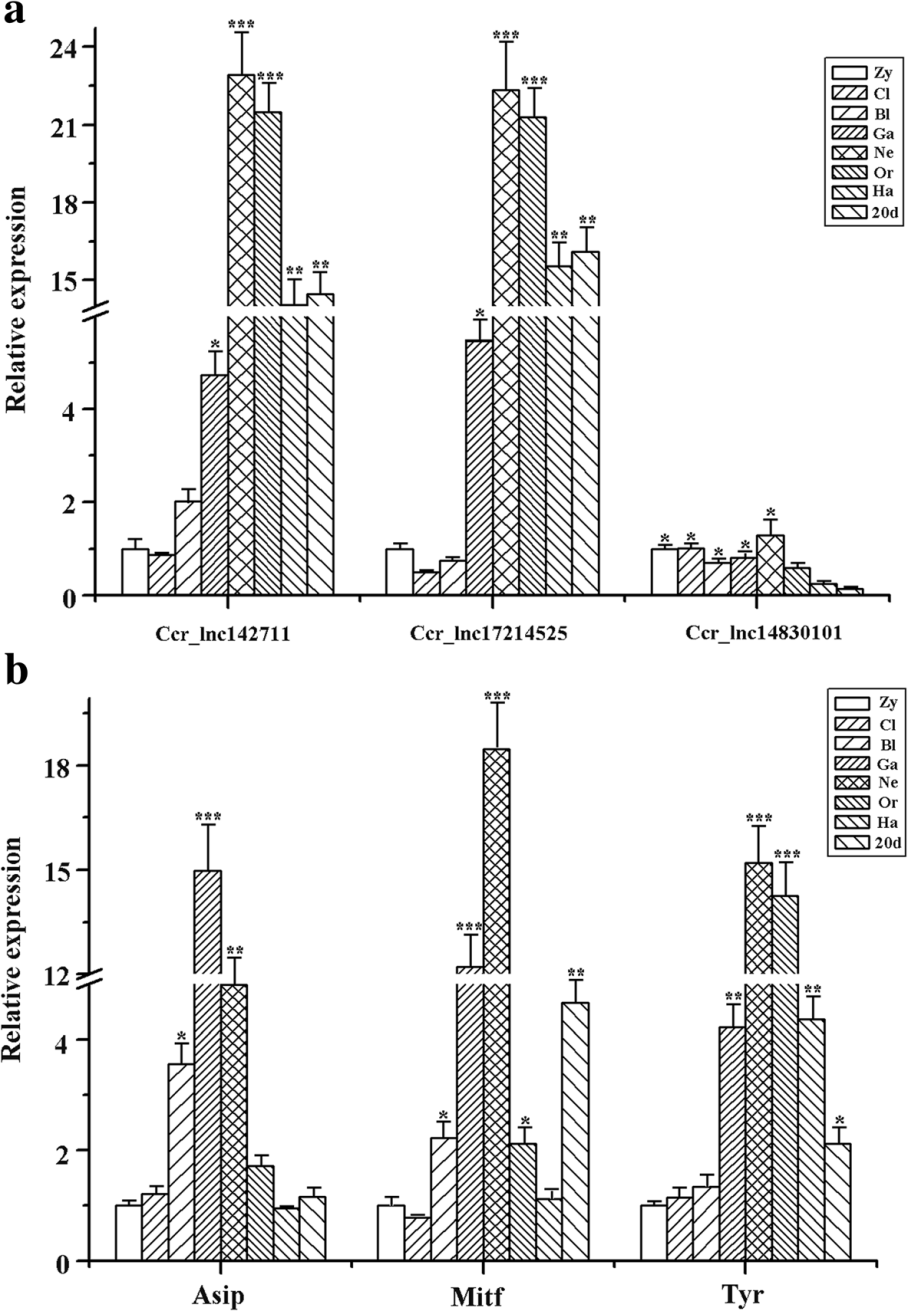
Overview of expression pattern of three lncRNAs (**a**) and three mRNAs (**b**) involved in melanogenesis pathway during the early stage of Koi carp. Zy, zygote; Cl, cleavage; BL, blastula; Ga, gastrula; Ne, neurula; Or, organogenesis; Ha, hatching; 20pdh, 20 days post-hatching.* *p* < 0.05, ** *p* < 0.01, *** *p* < 0.001

We also identified some key lncRNAs and target mRNAs related to the melanogenesis pathway (Table 3). The results showed that *Mitf* and *Wnt3a* genes, which are known to play crucial roles in skin color pigmentation, were linked to both *cis* and *trans* actions. Furthermore, we selected three lncRNAs (*Ccr_lnc142711*, *Ccr_lnc17214525* and *Ccr_lnc14830101*) and three mRNAs (*Asip*, *Mitf* and *Tyr*) to analyze their spatial and temporal expression characteristics. The results indicate that expression is tightly regulated in a time- and space-dependent manner. *Ccr_lnc142711*, *Ccr_lnc17214525*, *Asip* and *Tyr* were strongly expressed in gastrula, neurula and organogenesis stages, and *Mitf* expression was substantially up-regulated in gastrula and neurula stages, and after 20 days post-hatching (Fig. 8). In addition, we examined their expression profiles in different tissues of Koi carp. Interestingly, we found that *Ccr_lnc142711* and *Ccr_lnc148301* abundantly expressed in kidney, brain, blood, eye and gut, whereas *Ccr_lnc17214525* abundantly expressed in white skin and eye tissue (Additional file 8: Figure S3A). Expression of *Asip* was up-regulated in white skin, red skin, kidney, brain, eye, gonad and gut, and *Mitf* highly expressed in kidney, blood, eye and gut, whereas *Tyr* is abundantly expressed in black skin, eye and muscle (Additional file 8: Figure S3B).

**Table 3:** LncRNAs and their potential target genes involved in melanogenesis

Protein-coding genes	lncRNAs in *cis*	lncRNAs in *trans*
*Mitf*	*Ccr_lnc17821911*	*Ccr_lnc125640361, Ccr_lnc1485701, Ccr_lnc5622421, Ccr_lnc17214525, Ccr_lnc8963611*
*Wnt3a*	*Ccr_lnc8247101*	*Ccr_lnc105029701, Ccr_lnc1134941511, Ccr_lnc1442012, Ccr_lnc2059601, Ccr_lnc6033401, Ccr_lnc105029701*
*Tyr*		*Ccr_lnc17214525, Ccr_lnc14830101, Ccr_lnc8963611*
Tyrp1		*Ccr_lnc16301123, Ccr_lnc5622441, Ccr_lnc8963611, Ccr_lnc822721141*
*Asip*		*Ccr_lnc1485701, Ccr_lnc17214525, Ccr_lnc142711, Ccr_lnc113494151, Ccr_lnc11354001*, *Ccr_lnc8963611*
*Sox10*		*Ccr_lnc125640361, Ccr_lnc1485701, Ccr_lnc5622421, Ccr_lnc7090801, Ccr_lnc5622421,Ccr_lnc17214525*
*Tyk2*		*Ccr_lnc1134941511, Ccr_lnc11354001, Ccr_lnc15278331, Ccr_lnc11354001, Ccr_lnc12754831, Ccr_lnc12757551, Ccr_lnc131643581, Ccr_lnc14074601, Ccr_lnc14419221, Ccr_lnc14420121, Ccr_lnc14420301, Ccr_lnc147974311, Ccr_lnc17632401, Ccr_lnc175284642, Ccr_lnc3111401*

## Discussion

Koi carp are among the most popular ornamental fish worldwide, the body color and skin pattern is closely correlated with market value [36]. Thus, the mechanism of skin color regulation has been investigated, including transcriptomic analysis of pigment-associated genes [37, 38]. In our previous study, we identified important miRNAs (e.g. *miR-196a*, *miR-125b*, *miR-200b* and *miR-206*) that influence pigmentation and differentiation in the three main skin colors [23]. Despite an abundance of literature on tissue-specific expression patterns and functions of mRNAs and miRNAs in fish, evidence for tissue-restricted expression of lncRNAs is limited. However, in plants and non-fish animals, evidence suggests that some lncRNAs display strict cell specificity and play a key role in development and differentiation of tissues [39, 40]. Therefore, in an effort to better understand the influence of lncRNAs on mRNA functions and related regulatory mechanisms in Koi carp, we elucidated lncRNA profiles of three different phenotypes involved in skin pigmentation and differentiation using Illumina sequencing technology.

In the current study, 446,614 putative transcripts were obtained, including 4252 known and 72,907 novel lncRNAs, tremendously enriching the pool of lncRNAs correlated with fish skin. Expression of lncRNAs can be tissue-specific, and differential expression is widespread in a variety of organisms [41–43]. To gain insight into the expression of skin color-related genes in Koi carp, we determined expression profiles, and identified 92 DE lncRNAs and 722 DE mRNAs through pairwise comparison. Interestingly, levels of *tyr*, *pmela*, *pmelb* and *sex determining region Y-box 10* (*sox10*) were high in black skin compared with red and white skin, which implied that these genes contribute to black coloration. These expression trends and characteristics are similar to those in tilapia and other vertebrate species [44, 45]. We also identified some specific lncRNAs such as *Ccr_lnc5622441* and *Ccr_lnc765201* that were up-regulated in RS and BS groups compared to WS, while *Ccr_lnc14074601* and *Ccr_lnc2382951* were up-regulated in WS. However, more integrative experiments are needed to further explore their functional mechanisms related to skin color regulation. Verification by qRT-PCR confirmed the reliability of the RNA-seq data and the lncRNAs and mRNAs identified. Additionally, several previous studies support our results. For example, we found that expression of microphthalmia-associated transcription factor (*mitf*) was higher in BS than WS, and a previous study confirmed that *mitf* is a member of the basic helix-loop-helix leucine zipper (bHLH-Zip) protein family and directly regulates the differentiation of melanin cells by controlling the expression of tyrosine gene families [46, 47].

LncRNAs can function through *cis*-regulation of nearby protein-coding genes [48]. Only a few lncRNAs have been defined as *cis*-regulators, and most functionally annotated lncRNAs are *trans*-regulators [49]. In our study, DE lncRNAs were divided into two categories (*cis* or *trans*-regulatory genes) to explore potential target mRNAs. The results revealed that 70 lncRNAs corresponded to 107 protein-coding genes via *cis* regulation, and 79 lncRNAs corresponded to 41,625 protein-coding genes through *trans* regulation*.* Notably, *Mitf* and *Wnt3a*, both members of the Wnt family, were found to be regulated by lncRNAs both in *cis* (*Ccr_lnc17821911*) and in *trans* (*Ccr_lnc125640361*, *Ccr_lnc1485701* and others) in the melanogenesis pathway. These findings indicate that lncRNAs are highly functionally conserved, similar to their target Wnt signaling protein [50], although their regulatory mechanisms need to be further investigated. In addition, a number of lncRNAs acting in *trans* were found to target protein-coding genes specifically expressed in melanocytes, including *Tyr*, *Tyrp1*, *P450* and *Sox10*. For example, *Ccr_lnc8963611* acts on both *Tyr* and *Tyrp1*, which evolved from a common ancestral tyrosinase gene [51, 52]. A third interesting observation is that some mRNAs (*Mitfa*, *Slc7a11* and others) are regulated by multiple lncRNAs (e.g. *Ccr_lnc8963611* and *Ccr_lnc1485701*), and a single lncRNA (*Ccr_lnc5622421*) can target many mRNAs (*Mc1r* and *Tyrp1*). This indicates the functional complexity of lncRNAs. Therefore, we screened some DE *cis*- and *trans*-acting lncRNAs that function on genes related to pigmentation. However, these putative connections and interactions are based on theoretical analysis, and experimental verification is required.

Some pathways related to pigmentation have been identified, including cAMP [53], melanogenesis [54], Wnt [55], SCF-KIT [56], Notch [57], FGF/MAPK/Ets [58], MITF-GPNMB [59], CREB/MITF/Tyrosinase [60] and protein kinase C signaling pathway [61]. However, information on the roles of lncRNAs in pigmentation and differentiation in fish remains scarce. Methods for lncRNA function prediction can correlate protein-coding genes and their related biological pathways [62]. In our present study, we analyzed these *cis* target genes of DE lncRNAs enriched in 91 KEGG pathways, and the *trans* target genes enriched in 355 KEGG pathways. Some key pathways associated with pigmentation are enriched, including Wnt, cAMP and melanoma signaling pathways, as well as tyrosine metabolism. This strongly indicates that these pathways play essential roles in determining the three different skin colors.

To gain insight into the functions of some lncRNAs and mRNAs in physiological processes, we further analyzed their temporal and spatial expression patterns. The results indicate that *Ccr_lnc142711*, *Ccr_lnc17214525*, *Asip* and *Tyr* strongly expressed in gastrula, neurula and organogenesis stages. LncRNAs expressed during blastula and gastrula stages might play important roles in cell fate decisions, differentiation and cell migration [63]. In addition, pigment cells are initially derived from the neural crest during gastrula stage [64, 65]. Indeed, recent large-scale knockdown analyses in mouse embryonic stem cells (ESCs) revealed key roles for lncRNAs in cell fate specification [66]. Therefore, we speculated that *Ccr_lnc142711* and *Ccr_lnc17214525* may be potential regulators of the pigmentation process.

Deeper molecular mechanistic research is clearly needed to elucidate the specific functions lncRNAs and their target genes. To this end, we found that *Ccr_lnc142711* and *Ccr_lnc148301* abundantly expressed in kidney, brain, blood, eye and gut, whereas *Ccr_lnc17214525* abundantly expressed in white skin and eye tissues, indicating strict cell specificity for lncRNAs [39]. Skin pigmentation in fish is a complex process that involves numerous genetic factors [67]. Herein, it is found that expression of *Asip* is up-regulated in white skin, red skin, kidney, brain, eye, gonad and gut, while *Mitf* highly expressed in kidney, blood, eye and gut, and *Tyr* abundantly expressed in black skin, eye and muscle. The results are similar to some previous research [68–70], which suggest that expression of these genes is associated with skin pigmentation.

## Conclusion

To our knowledge, this study provides the first lncRNA-mRNA integrated profiling analysis of differences in skin color in Koi carp. By comparing RNA-seq data from three pairwise groups, we identified significant DE lncRNAs and mRNAs, and explored potential *cis* and *trans* targets of lncRNAs. Functional enrichment and lncRNA-mRNA regulatory network analyses revealed novel lncRNAs and mRNAs associated with pigmentation and differences in skin color. Additionally, temporal and spatial expression patterns of lncRNAs and mRNAs revealed tissue specificity. Our results provide new insight into the molecular mechanisms underlying lncRNA-mediated pigmentation and differentiation in Koi carp. The numerous lncRNAs and mRNAs identified, and the accompanying de novo transcriptome data, provide valuable resources for future transcriptome studies investigating functions associated with skin color in fish.

## Methods

### Sample collection

Koi carp were obtained from the Qiting Pilot Research Station (Yixing, Jiangsu, China), affiliated to the FFRC. Animals (average weight: 400 ± 8 g) were raised at 24 ± 1 °C in 256 L tanks in a circulation water system for 1 week before experiments and fed twice daily with compound feed (Tech-bank Co., Ltd., Ningbo, China). Aeration was supplied constantly and a 12 -h light / dark photoperiod was employed.

Fish in the three experimental groups (black, white and red spots in one fish synchronous; Additional file 1: Figure S1) were tranquilized with 10–15 mg/L MS-222 buffered to pH 7.0–7.5, and three sets of skin tissues, black (BS), white (WS) and red (RS), were collected, immediately snap-frozen in liquid nitrogen and stored at − 80 °C until RNA isolation. After skin tissues sampling, fish were euthanized in buffered 300 mg/L Methyl methanesulfonate (MMS) and kidney, heart, eye, gill, gut, brain, blood, muscle, liver and gonad tissues were also collected and conserved at − 80 °C until use.

Samples from different developmental stages were collected at the aquaculture base. Brood fish were processed under hormone stimulation and mass spawning conditions. Fertilized eggs were hatched in cement tank with constant aeration at 24 ± 1 °C. Developmental stages were divided into zygote, cleavage, blastula, gastrula, neurula, organogenesis, hatching and 20 days post-hatching as described previously [24]. Samples from the same developmental stage were collected, immediately snap-frozen in liquid nitrogen and stored at − 80 °C until use.

### RNA isolation, library preparation and sequencing

Total RNA was isolated using TRIzol reagent (Invitrogen, Carlsbad, CA, USA) according to the manufacture’s protocol, and genomic DNA was removed using DNase I (New England Biolabs). The concentration and integrity of RNA were estimated using a NanoDrop 2000 (Thermo Scientific, USA) and RNA quality was confirmed with an Agilent 2100 Bioanalyzer (Agilent Technologies, CA, USA).

Poly(A) libraries were prepared using Illumina TruSeq RNA Sample Preparation Kit v2 (NEB, Ipswich, MA, USA). Poly(A) RNA was purified with oligo dT magnetic beads, then fragmented with divalent cations followed by reverse transcription into cDNA and ligation of Illumina paired-end oligo adapters to cDNA fragments. Next, nine sequencing libraries were constructed and each library was loaded into one lane of the Illumina Hi-Seq X-ten for 150 bp paired-end sequencing.

### Bioinformatics analysis

The pipeline for lncRNA analysis presented in (Additional file 1: Figure S2).

#### Quality control

FastQC (http://www.bioinformatics.babraham.ac.uk/projects/fastqc/) [25] was used to perform quality control of the sequencing data. Poor quality reads, including reads with adaptors, reads with > 10% unknown bases, and low-quality reads with > 50% bases with a quality value ≤5 were discarded during the initial filtering step.

#### Transcriptome assembly

Clean reads were first mapped onto the common carp (*C. carpio*) v3.0 reference genome (http://www.fishbrowser.org/database/Commoncarp_genome/) independently by TopHat v2.0.10 [26] with six mismatches prior to assembly. Clean reads were then de novo assembled into transcripts using a genome-guided method by Trinity v2.4.0 [27] with default parameters to reconstruct transcripts based on the actual read sequences. Finally, the obtained non-redundancy assembled transcripts were clustered using CD-HIT v4.6 [28] for downstream analysis.

#### Novel LncRNA prediction

Firstly, we used blat (https://genome.cshlp.org/content/12/4/656.short) to align assembled transcripts against the reference genome to identify locations of assembled transcripts with at least 90% sequence similarity. Since no lncRNA information was provided in *C. carpio* v3.0 genome annotations, transcripts similar to known lncRNAs in *Danio rerio* NONCODE v5 (cutoff E-value ≤1e-10) were assigned as putative lncRNAs.

In order to idnetify novel lncRNAs from the final assemblies, transcript locations were compared with reference genome annotation using Gffcompare (v0.10.1, https://ccb.jhu.edu/software/stringtie/gffcompare.shtml). We then followed the steps below to filter novel lncRNAs from the newly identified transcripts based on transformed locations:

1. Only ‘u’ category transcripts were considered to contain novel lncRNAs;
2. According to most studies, putative lncRNAs are arbitrarily defined as transcripts ≥200 nt in length with no protein-coding ability, hence transcripts < 200 nt were filtered [29];
3. Transcripts overlapping with known lncRNAs in *Danio rerio* were filtered;
4. More than 95% protein-coding genes have open reading frames (ORFs) > 100 amino acids [30]. We used TransDecoder version rel16JAN2014 (https://sourceforge.net/projects/transdecoder/) to identify putative ORFs in each transcript, and removed transcripts with putative ORFs > 300 bp;
5. We aligned the remaining transcripts to the NCBI non-redudant (Nr) protein database to eliminate transcripts similar to known proteins (cutoff E-value ≤1 × 10^− 5^);
6. Coding Potential Calculator version 0.9-r2 (http://www.mybiosoftware.com/cpc-0-9r2-assess-protein-coding-potential-transcripts.html) was utilized to estimate the coding potential of each transcript using the complete UniRef90 database with default parameters. Sequences with protein-coding-scores (CPC-score) < 0 were classified as novel lncRNAs.

#### Identification of differentially expressed lncRNAs and mRNAs

To analyze transcript abundance in a genome-free manner, RSEM v1.2.22 [31] was employed to estimate and quantify gene expression using an alignment-based method, yielding an expected read count for each protein and lncRNA gene. Gene expression was normalized using the fragments per kilobase of exon per million reads mapped (FPKM) method. Finally, edgeR v3.22.3 [32] was used to identify differentially expressed genes by pairwise comparisons. Differences were considered significant for FDR ≤0.05 and |log2 fold change (FC)| ≥1. Differential cluster analysis of genes and volcano plotting of differentially expressed lncRNAs and mRNAs were performed using the E Charts platform (http://www.ehbio.com/ImageGP/index.php/Home/Index/index.html).

#### Target gene prediction

The *Cis* role refers to the action of lncRNAs’ on neighboring target genes. We searched for coding genes within 10–100 kb upstream or downstream of lncRNAs.

The *Trans* role represents the influence of lncRNAs on other genes at the expression level. We calculated the expressed Pearson correlation coefficient between lncRNAs and corresponding coding genes using custom scripts (*p* < 0.05 and R ≥ 0.95). A regulatory network was then constructed and visualized by Cytoscape v3.6.1 [33], a standard tool for integrated analysis and visualization of biological networks.

#### GO and KEGG pathway analysis

To explore the functions of significant differentially expressed lncRNAs and mRNAs, and the corresponding target genes of differentially expressed lncRNAs, GO term and KEGG pathway enrichment analysis were conducted using the DAVID program v6.8 (https://david.ncifcrf.gov/) [34]. Significance was expressed as *p*-value, with a lower *p*-value indicating higher significance.

### Quantitative real-time PCR assay

Total RNA from different tissues was extracted as described above, 0.5 μg was reverse-transcribed into first-strand cDNA using a PrimeScript RT Reagent Kit (TaKaRa, Japan), and qRT-PCR was performed on a CFX96 Touch Detection System (Bio-Rad, Hercules, CA, USA) using SYBR Premix Ex Taq II reagent (TaKaRa). All reactions were conducted in triplicate for each sample with an initial denaturation at 95 °C for 30 s, followed by 40 cycles at 95 °C for 5 s, and the optimized annealing temperature for 30 s. The relative expression levels of differentially expressed lncRNAs and mRNAs were normalized against glyceraldehydes-3-phosphate dehydrogenase (*GAPDH*) and *β-actin* using the 2^-△△Ct^ method [35]. All primers (Table S1, Additional file 1) were designed using Primer Premier 5.

### Statistical analysis

Sequencing data were analyzed as described above. All other data are presented as mean ± standard error of the mean (SEM) and were calculated by SPSS 22.0 (SPSS Inc., Chicago, IL, USA) using one-way analysis of variance (ANOVA) followed by Duncan tests (*p* < 0.05 = significant and *p* < 0.01 = highly significant).

## Additional files

Additional file 1: **Figure S1.** Photograph showing the three different skin color types in Koi carp. **Table S1.** Primers used for analysis of differentially expressed genes related to skin color in Koi carp. **Figure S2.** Pipeline for analysis of skin color-related lncRNAs by Illumina sequencing. (DOCX 260 kb)

Additional file 2: Quality control results of nine representative samples. (XLSX 10 kb)

Additional file 3: Known and novel lncRNAs identified in black, white and red skin samples from Koi carp. (XLSX 3809 kb)

Additional file 4: Differentially expressed lncRNAs and mRNAs identified in the three different skin color groups. (XLSX 136 kb)

Additional file 5: GO term and KEGG pathway enrichment analysis of significant differentially expressed mRNAs in the three skin color groups. (XLSX 304 kb)

Additional file 6: Potential *cis* targets of lncRNAs with GO term and KEGG pathway enrichment analysis. (XLSX 51 kb)

Additional file 7: Potential *trans* targets of lncRNAs with GO term and KEGG pathway enrichment analysis. (XLSX 28106 kb)

Additional file 8: Expression profiles of three lncRNAs (A) and three mRNAs (B) involved in the melanogenesis pathway in different tissues of Koi carp. (DOCX 494 kb)

## Data Availability

All raw transcriptome data reported in this article have been deposited in the NCBI and Sequence Read Archive (SRA) databases (https://www.ncbi.nlm.nih.gov) under accession numbers SRR8281645, SRR8281646, SRR8281647, SRR8281648, SRR8281649, SRR8281650, SRR8281651, SRR8281652 and SRR8281653. Sample metadata expression estimates can be found on the NCBI Gene Expression Omnibus under accession number GSE125039.

## Abbreviations

CAFS: Chinese Academy of Fishery Sciences
cAMP: Cyclic adenosine monophosphate
cDNA: Complementary DNA
CPC: Coding Potential Calculator
FFRC: Freshwater Fisheries Research Center
Fgf: Fibroblast growth factor
GAPDH: Glycer-aldehyde-3-phosphate dehydrogenase
GO: Gene Ontology
KEGG: Kyoto Encyclopedia of Genes and Genomes
LncRNAs: Long non-coding RNAs
MAPK: Mitogen-activated protein kinase
Mc1r: Melanocortin receptor 1
Mitf: Microphthalmia-associated transcription factor
NCBI: National Center for Biotechnology Information
NcRNAs: Non-coding RNAs
Notch: Notch signaling pathway
Nr: Non-redudant
ORFs: Open reading frames
Pmela: Premelanosome protein a
Pmelb: Premelanosome protein b
QRT-PCR: Quantitative real-time PCR
Silv: Silver locus protein homolog
Sox10: Sex determining region Y-box 10
sRNA-Seq: Small RNA sequencing
Tyr: Tyrosinase
Tyrp1: Tyrosine related protein-1
Wnt: Wingless
