# Supplementary material for: Integrated analysis of long non-coding RNA and mRNA expression in different colored skin of koi carp
Source: BMC Genomics. 2019 Jun 21;20:515. doi: 10.1186/s12864-019-5894-8 (PMC6588874; doi:10.1186/s12864-019-5894-8)
Supplement: Supplementary file 1 — Figure S1. Photograph showing the three different skin color types in Koi carp. Table S1. Primers used for analysis of differentially expressed genes related to skin color in Koi carp. Figure S2. Pipeline for analysis of skin color-related lncRNAs by Illumina sequencing. (DOCX 260 kb) [file 12864_2019_5894_MOESM1_ESM.docx]

**
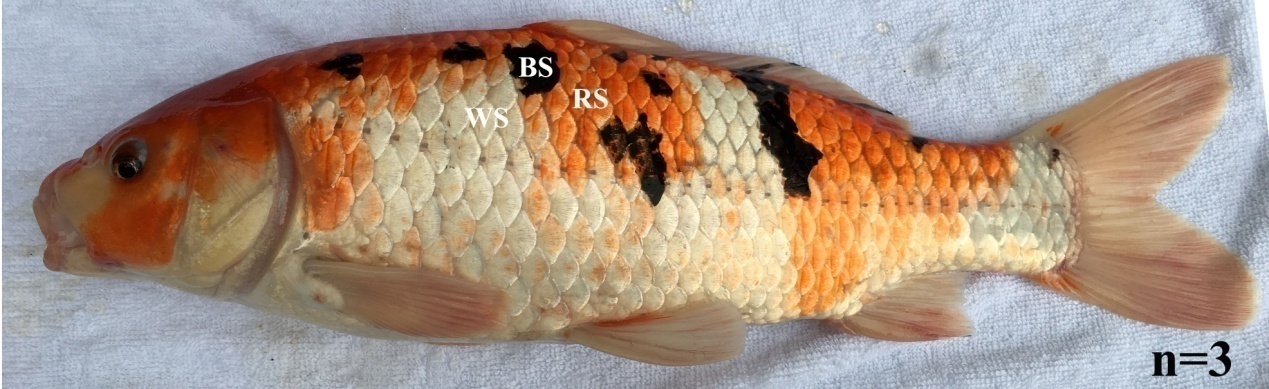
**

**Figure S1. Photograph showing the three different skin color types in Koi carp. RS, red skin; BS, black skin; WS, white skin; n, sample number**

**
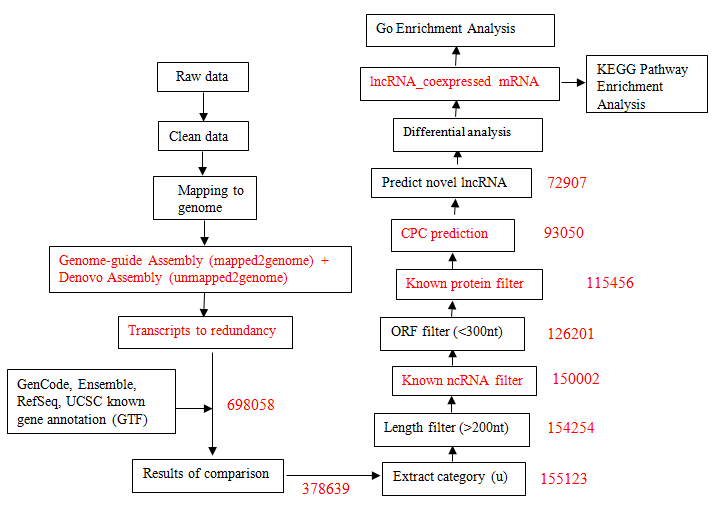
**

**Figure S2. Pipeline for analysis skin color-related lncRNAs by Illumina sequencing**

**Table S1. Primers used for analysis of differentially expressed genes related to skin color in Koi carp**

| Genes | Forward primers (5’-3’) | Reverse primers (5’-3’) |
| --- | --- | --- |
| *Ccr_lnc17214525* | TATTTTGAAGAGCGGGGATG | GCTGAAGTTGATTGATGCGA |
| *Ccr_lnc14830101* | CACACTGCCCCGTAGAAGAT | ATATTCCGCCAGTGAACGAG |
| *Ccr_lnc105029701* | TATGTGACAACTCCGCGCTT | CAGCTCAACACAAGCCGTTC |
| *Ccr_lnc8963611* | CACGGTATGGCACACAGGTA | GGACTGTTAGCGCTTGGTTC |
| *Ccr_lnc1485701* | CGCCCACAGTTAAATCCACT | ACCTACTGGACTGCCCAATG |
| *Ccr_lnc5622451* | ATGAAACGGGACTGGAGTTG | ACCAACCCAGTGACTCCAAG |
| *Ccr_lnc13164358* | AGAATAGCCGTTCGGTTA | TTGTCACGAGGCAGTTTACA |
| *Ccr_lnc16063311* | TGTTTGTGGTGTCGGCAG | AGTGCAGGAGAGCCACTA |
| *Ccr_lnc17821911* | ACCCCAAGGTTCCAATTTTC | GGGGTCTCAGTCAAACCTGA |
| *Ccr_lnc142711* | CGCCCACAGTTAAATCCACT | ACCTACTGGACTGCCCAATG |
| *Foxd3* | AACCGCTTCCCTTACTACCG | GAGTAAGCGGCAGGGTGAAT |
| *Slc7a11* | ACACGTACATTCTGGAGGCG | ACCCAAGCCAAAGCGTACATC |
| *Asip* | AACTGCGTGCCGCTCTTGA | TAAACAAGCCTTTGGGATCGG |
| *Tyrp1* | CGATGGCCACTTCGGTTCTTTA | CTGTACACTTGCTCCGTCCG |
| *Cbs* | GGCCCACTATGACACCACAG | AAGCCCTTATCACACATCCAG |
| *Mitf* | CGAAGTACCACATCCAGCAGAC | TTGTCCATGAGCATGTCCTCCA |
| *Tyr* | CACGGTCTCCGATCTTCCC | CATCACGCCAGTCCCAGTA |
| *Dct* | CCCTGCGTTCATCACCTG | CTGTCACACACCACTCCCC |
| *Sox10* | AAAACTCTGGAGGCTGCTGAA | TTGTAATGCGATTGGCTGTGA |
| *Mlph* | GGCAGCGTAGGAATGTCACT | ACCCAAGCCAAAGCGTACA |
| *GAPDH* | AATTTCCTTCCAGGCATGTG | GCAACCGTGTATGTGACCTG |
| *β-actin* | CGTGATGGACTCTGGTGA | ACAGTGTTGGCATACAGGT |
