# Supplementary material for: Integrated analysis of long non-coding RNA and mRNA expression in different colored skin of koi carp
Source: BMC Genomics. 2019 Jun 21;20:515. doi: 10.1186/s12864-019-5894-8 (PMC6588874; doi:10.1186/s12864-019-5894-8)
Supplement: Supplementary file 8 — Expression profiles of three lncRNAs (A) and three mRNAs (B) involved in the melanogenesis pathway in different tissues of Koi carp. (DOCX 494 kb) [file 12864_2019_5894_MOESM8_ESM.docx]

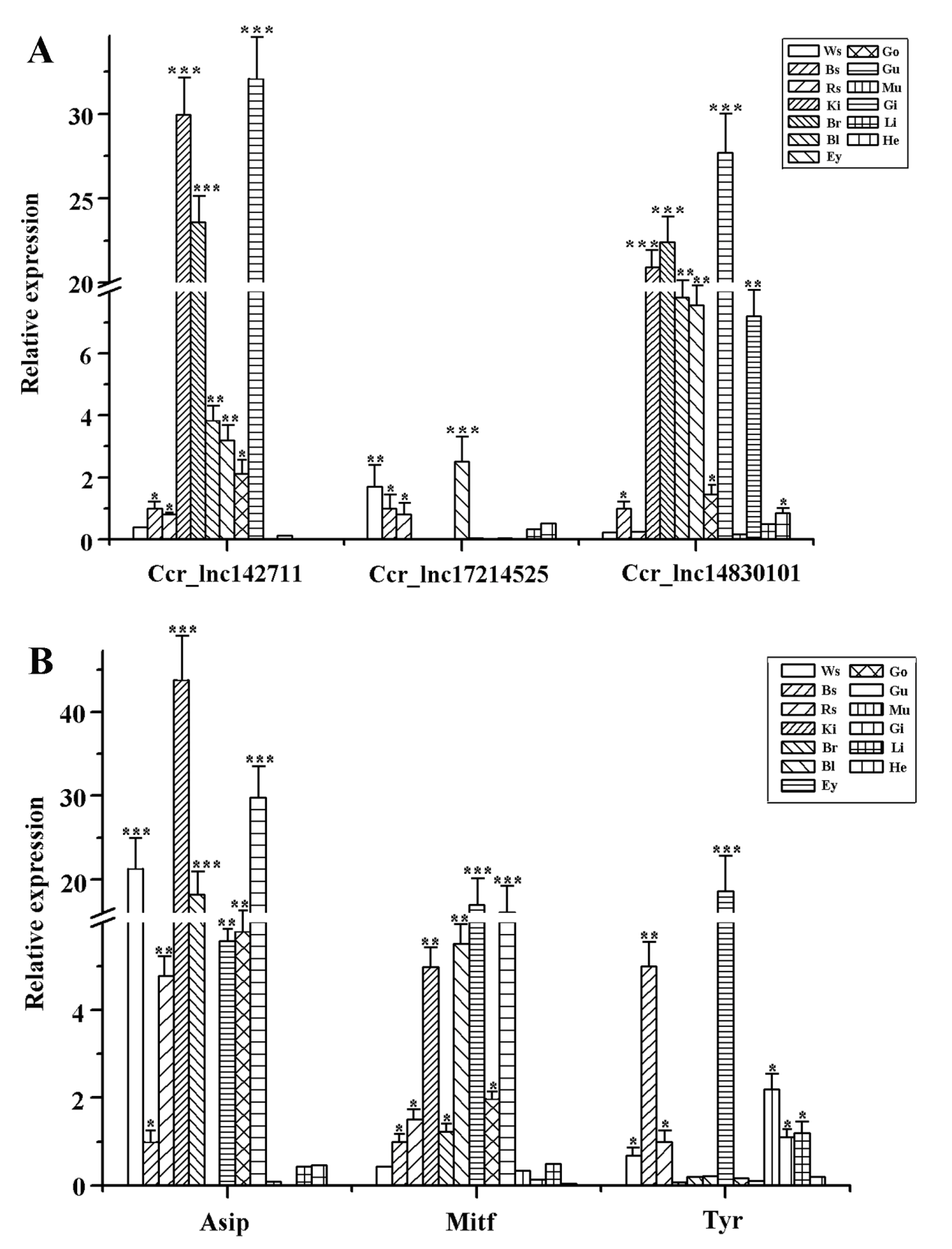


**Figure S3. Expression profiles of three lncRNAs (A) and three mRNAs (B) involved in the melanogenesis pathway in different tissues of Koi carp. Ws, white skin; Bs, black skin; Rs, red skin; Ki, kidney; Br, brain; Bl, blood; Ey, eye; Go, gonad; Gu, gut; Mu, muscle; Gi, gill; Li, liver; He, heart. * *p* <0.05, ** *p* <0.01, *** *p* <0.001.**
